# In vivo imaging reveals sigmoidal growth kinetic of β-amyloid plaques

**DOI:** 10.1186/2051-5960-2-30

**Published:** 2014-03-28

**Authors:** Steffen Burgold, Severin Filser, Mario M Dorostkar, Boris Schmidt, Jochen Herms

**Affiliations:** 1 German Center for Neurodegenerative Diseases (DZNE), Ludwig-Maximilians-University Munich, Feodor-Lynen-Str. 23, 81377 Munich, Germany; 2 Center for Neuropathology, Ludwig-Maximilians-University Munich, Feodor-Lynen-Strasse 23, 81377 Munich, Germany; 3 Munich Cluster of Systems Neurology (SyNergy), Ludwig-Maximilians-University Munich, Schillerstrasse 44, 80336 Munich, Germany; 4 Clemens Schoepf-Institute for Organic Chemistry and Biochemistry, Technische Universität Darmstadt, Petersenstrasse 22, 64287 Darmstadt, Germany

**Keywords:** Alzheimer’s disease, β-amyloid, Aggregation kinetics, Plaque growth, Two-photon imaging, In vivo

## Abstract

A major neuropathological hallmark of Alzheimer’s disease is the deposition of amyloid plaques in the brains of affected individuals. Amyloid plaques mainly consist of fibrillar β-amyloid, which is a cleavage product of the amyloid precursor protein. The amyloid-cascade-hypothesis postulates Aβ accumulation as the central event in initiating a toxic cascade leading to Alzheimer’s disease pathology and, ultimately, loss of cognitive function. We studied the kinetics of β-amyloid deposition in Tg2576 mice, which overexpress human amyloid precursor protein with the Swedish mutation. Utilizing long-term two-photon imaging we were able to observe the entire kinetics of plaque growth *in vivo*. Essentially, we observed that plaque growth follows a sigmoid-shaped curve comprising a cubic growth phase, followed by saturation. In contrast, plaque density kinetics exhibited an asymptotic progression. Taking into account the fact that a critical concentration of Aβ is required to seed new plaques, we can propose the following kinetic model of β-amyloid deposition *in vivo*. In the early cubic phase, plaque growth is not limited by Aβ concentration and plaque density increases very fast. During the transition phase, plaque density stabilizes whereas plaque volume increases strongly reflecting a robust growth of the plaques. In the late asymptotic phase, Aβ peptide production becomes rate-limiting for plaque growth. In conclusion, the present study offers a direct link between *in vitro* and *in vivo* studies facilitating the translation of Aβ-lowering strategies from laboratory models to patients.

## Introduction

Alzheimer’s disease (AD) is the most common form of dementia and is characterized by the accumulation of amyloid plaques as one major neuropathological hallmark [1]. Amyloid plaques consist mainly of Aβ peptide (Aβ) that is produced by sequential cleavage of amyloid precursor protein (APP) by β-secretase and γ-secretase [2]. The amyloid-cascade-hypothesis postulates that Aβ accumulation is the central event in the etiology of AD, initiating a toxic cascade that culminates in widespread neurodegeneration [3-6]. Indeed, recent biomarker studies in humans have shown that changes in Aβ metabolism and Aβ accumulation initiate about 20 years before the onset of clinical symptoms [7-10].

Cloning of the amyloid precursor protein pioneered the investigation of Aβ aggregation *in vitro*[11]. Monomers of Aβ were shown to form higher order aggregates in a time and concentration dependent manner [12]. The process of *in vitro* Aβ aggregation, as well as Aβ accumulation in humans, with time follows a sigmoidal curve shape [7,13,14].

In recent years several studies investigated the kinetics of Aβ aggregation into amyloid plaques in AD mouse models [15]. Collectively, these studies revealed that newly formed plaques were initially very small and enlarged slowly over long periods of time during the early stages of amyloid pathology [16-20], while late-stage amyloid pathology lacked further plaque formation [17,19,21] and growth [16,17,20,22].

In the present study we aimed at extending this basic knowledge of *in vivo* plaque growth kinetics by investigating the close relationship between plaque density and plaque growth. Furthermore, we were able to monitor the complete plaque growth kinetics over an observation period of 15 months that reflect both the early and late stage amyloid pathology. In this manner, we found that plaque growth follows an initial cubic and a late asymptotic phase.

## Materials and methods

### Transgenic mice

Heterozygous Tg2576 [9] mice (B6;SJL-Tg(APPSWE)2576Kha from Taconic, Cologne, Germany) were kindly provided by neuroanatomy group of Abbott (Ludwigshafen, Germany). Tg2576 mice express a human APP with the Swedish mutation (K670M/N671L) under a hamster prion protein promoter. Heterozygous APPPS1 mice coexpress a human APP with the Swedish mutation and a mutated PS1 (L166P) under the neuron-specific Thy1-promoter [23]. Mice were of both sexes (four females in the young cohort and one male/three females in the old cohort) and group-housed under pathogen-free conditions until surgery, after which they were single-housed. All procedures were performed in accordance with an animal protocol approved by the University of Munich and the Government of Upper Bavaria [Az. 55.2-1.54-2531-110-06].

### Cranial window surgery

A cranial window was implanted over the right cortical hemisphere as previously reported [24-27]. In short, the mice were anesthetized with an intraperitoneal injection of ketamine/xylazine (0.13/0.01 mg/g body weight; WDT/Bayer Health Care, Garbsen/Leverkusen, Germany). Additionally, dexamethasone (0.02 ml at 4 mg/ml; Sigma) was intraperitoneally administered immediately before surgery [28]. A circular piece of the skull (about 5 mm in diameter) over the right hemisphere (centered over the parietal bone, approx. 5.5 mm caudal from the bregma and 5.5 mm lateral from midline) was removed using a dental drill (Schick-Technikmaster C1; Pluradent; Offenbach, Germany). The craniotomy was closed immediately with a round coverslip (5 mm in diameter), held with dental acrylic. A small metal bar, containing a hole for a screw, was glued next to the coverslip to allow repositioning of the mouse during subsequent imaging sessions. After surgery, mice received subcutaneous analgesic treatment with carprophen (7.5 mg/kg body weight; Rimadyl; Pfizer, New York, USA) and antibiotic treatment with cefotaxim (0.25 mg/g body weight; Pharmore, Ibbenbüren, Germany).

### Long-term two-photon in vivo imaging

Imaging started 3 to 4 weeks after the cranial window preparation to allow the animals to recover from surgery. For amyloid staining methoxy-X04 [29] was intraperitoneally injected 24 h before imaging. Initially, we administered a loading dose of 2 mg/kg body weight and in subsequent weekly imaging sessions a maintenance dose of 0.4 mg/kg body weight [17]. Two-photon imaging was performed on a LSM 7 MP (Zeiss, Jena, Germany) equipped with standard photomultiplier detectors and a 20x water-immersion objective (W Plan-Apochromat 20x/1.0 DIC, 1.0 NA, Zeiss, Jena, Germany). Methoxy-X04 was excited at 750 nm by a Ti:Sa laser (MaiTai DeepSee, Spectra-Physics, Darmstadt, Germany) and emission was collected from 440 to 500 nm. Image stacks of 850x850x250-400 μm^3^ were acquired using the “tilescan” mode of the microscope control software (Zen2009/Zen2010 64bit) that performs automatic stitching of several fields of view (2×2 were used) with a lateral resolution of 0.83 μm and 3 μm separation distance between consecutive images. Mice were anesthetized with isoflurane (Forene®, Abbott, Wiesbaden, Germany) for imaging and fixed to a custom-made holder using the glued metal plate. In subsequent imaging sessions, previously imaged volumes were identified by eye using the unique blood vessel pattern and fine adjusted by the positions of preexisting plaques. This allowed a precise alignment of the same imaging volume over a period of up to 15 months. The laser intensity was adjusted to keep the emitted fluorescence stable at different depths using the z-correction tool in the microscope control software and also at subsequent imaging sessions.

The following volumes were imaged: young cohort 2.888 mm^3^ (12 positions), old cohort 3.111 mm^3^ (16 positions), 12 to 18 months 1.076 mm^3^ (4 positions) and 12 to 27.5 months 0.559 mm^3^ (2 positions). The following numbers of plaques were analyzed for each imaging cohort: 50 newly formed and 101 preexisting plaques (12 to 14 months), 786 plaques (18 to 20 months) and 90 plaques (12 to 27.5 months).

### Image processing

The images with data depth of 12 bit were analyzed as time series of three-dimensional (3D) images in Imaris (Versions 6.2.1/7.4.2, Bitplane, Zurich, Switzerland). First, images were contrast-normalized (i.e., based on the average and standard deviation of intensities of 3D stacks). Plaque volumes were extracted by 3D-surface-rendering with background subtraction and a threshold of 500. All detected plaques were tracked by utilizing a custom-written Matlab plugin for Imaris or the surface tracking module of Imaris. Newly formed plaques were tracked back to the first time point when they appeared and were only assessed when present for at least 3 time points. Kinetic volume data were exported as Excel files. 3D stacks are either displayed as 3D-volume-rendered images (“normal shading” algorithm, Imaris) or as 3D-surface-rendered images in figures. Only the image data of detected plaques are shown in figures to focus the viewer’s eye on the essential information within the images. Background signals from autofluorescence were removed whereas contrast settings within time series were kept constant.

### Data analysis and statistics

For each plaque the radii from each time point were calculated from the kinetic volume data assuming a spherical shape of plaques [19]. Plaque densities were calculated for each imaged position. All other calculations, curve fitting and graphs were done in Prism (Version 5.04, GraphPad, La Jolla, USA). Data were tested for normality using D’Agostino-Pearson omnibus K2 test. Plaque densities and sizes had log-normal distributions and were therefore logarithmized which allowed for parametric statistical testing. These data were displayed with an “antilog” scale to represent the real measurement dimensions. For each plaque a linear growth rate was determined as the slope of a linear regression from radii calculations over time. Linear plaque growth rates were not normally distributed. All statistical tests are specified in the figure legends.

## Results

### Long-term in vivo imaging over 15 months reveals sigmoid-shaped plaque growth kinetics

**Figure 1:**
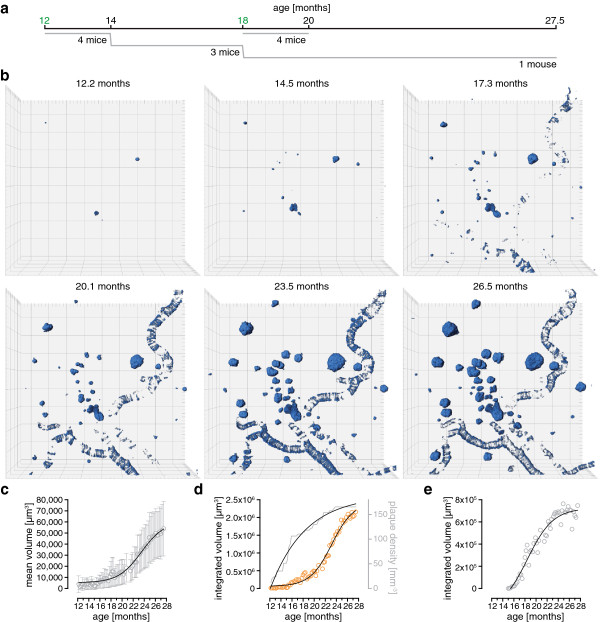
**Long-term *in vivo *imaging of amyloid plaque growth kinetics in Tg2576 mouse model of AD. (a)** A young and an old cohort were investigated from 12 to 14 and 18 to 20 months of age, respectively, at a weekly imaging intervals. **(b)** Time series of 3D volume-rendered images acquired with two-photon fluorescence microscopy showing amyloid plaques and cerebral amyloid angiopathy (CAA). The length of one side of the squares equals 100 μm. **(c)** Mean volumes of all plaques over time (open circles). The black line indicates a fitted sigmoid function (R^2^ 0.983). Error bars show the 95% confidence intervals (CI). (**d**) Integrated volume of all plaques over time (open circles). The black line indicates a fitted sigmoid function (R^2^ 0.988). In addition, the temporal development of the plaque density is depicted (gray line) which can be fitted to an exponential function of one phase association (R^2^ 0.975). **(e)** Integrated volume of the cerebral amyloid angiopathy (open circles) with the corresponding fit of a sigmoid function (R^2^ 0.952).

Tg2576 mice express human amyloid precursor protein with the Swedish mutation under the control of a hamster prion protein promoter [9], leading to the accumulation of Aβ-peptide and deposition of amyloid plaques starting at 8 to 10 months of age [9,30]. The fluorescent marker methoxy-X04 was injected [29] to visualize fibrillar amyloid through open-skull cranial windows over long time periods. We imaged two different cohorts starting at 12 and 18 months of age over at least 2 months, in weekly intervals (Figure 1a). These time-frames were chosen since plaque development is very dynamic in the young cohort whilst being more static in the older mice [17,22]. The mice from the young cohort were imaged for as long as technically feasible, limited by the clarity of the cranial window, in order to reconcile the measurements within both age cohorts in a single time series (Figure 1a). For precise measurement of the plaque density and in order to maximize the number of analyzable plaques, big volumes that typically sized 850 × 850× 250–400 μm^3^ (*x*, *y*, *z* dimensions) were scanned to 27.5 months (Figure 1b; see Additional file 1 for the complete time series and the Material and Methods section for all integrated volumes of each cohort). All images were of optimal quality and did not suffer from motion artifacts due to breathing or heart beating of the animal. The volume of plaques and cerebral amyloid angiopathy (CAA) were assessed by 3D surface-rendering and tracked over time. This analysis revealed a nonlinear sigmoid-shaped curve for the mean (R^2^ 0.983, Figure 1c) and also integrated volume (R^2^ 0.988, Figure 1d) of plaques. The integrated volume of the CAA followed a sigmoid function, too (R^2^ 0.952, Figure 1e). In contrast, the plaque density kinetics exhibited an asymptotic progression and could be fitted to a one-phase association function (R^2^ 0.975, Figure 1d).

**Figure 2:**
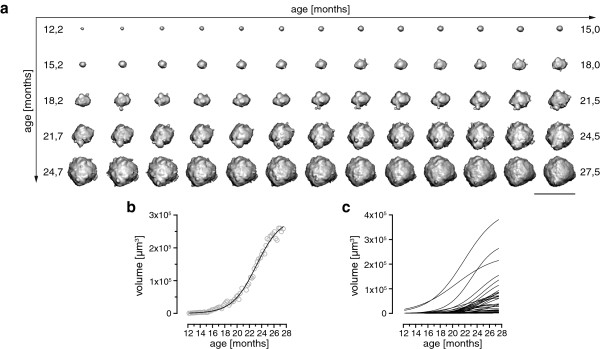
**Illustration of a single plaque over the whole imaging period of 15.5 months. (a)** Time series of a single plaque as a surface-rendered object as derived from 3D image analysis. Scale bar represents 100 μm. **(b)** Volume from the plaque shown in (a) over time (open circles) with the corresponding fit of a sigmoid function (black line). **(c)** Sigmoid fits of plaque volumes of all imaged plaques in one mouse. Note the considerable variance in plaque volumes.

Next, we analyzed the growth kinetics for each individual plaque separately, rather than the mean of all plaques as previously (Figure 2). We found that single plaque kinetics could also be fitted into a sigmoid function (Figure 2a,b). A comparison of all fitted curves for each individual plaque (originating from the example in Figure 1b) highlighted the fact that only a few plaques became very large whereas the majority of plaques showed much flatter curves with slower Aβ accumulation (Figure 2c).

### Quantification of plaque densities, sizes and growth rates in young and aged mice

**Figure 3:**
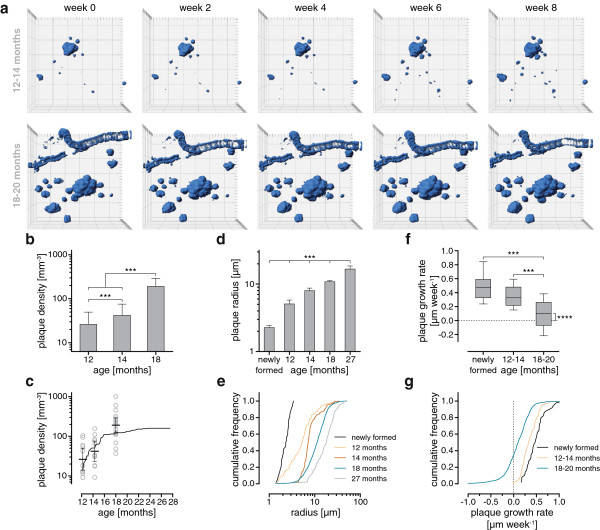
**Summary of plaque densities, radii and growth rates from all imaging cohorts. (a)** Representative examples of imaged volumes from the young and old cohort displayed as 3D volume-rendered images. The length of one side of the squares represents 50 μm. **(b)** Mean plaque densities from the young (12 and 14 months) and old cohorts (18 months). **(c)** The kinetic of plaque density from long-term imaging (mean from 2 positions) is shown as black line. In addition, plaque densities for each imaged position with their respective means from different cohorts are displayed at the corresponding age. **(d)** The mean plaque radius and their corresponding cumulative frequency distributions **(e)** are depicted for each imaging cohort including newly formed plaques from young cohort (two month imaging). For the long-term imaging cohort plaque sizes at 27 months are shown. **(f)** Box plot for linear plaque growth rates and **(g)** their corresponding cumulative frequency distributions. Plaque growth from 18 to 20 months of age was significant different from zero (P < 0.0001). Error bars show 95% CI **(b-d)**, Whiskers represent 10th and 90th percentile and outliers are not shown **(f)**. Plaque densities and radii were logarithmized before analysis **(b-e)**. Statistical tests: one-way ANOVA with Tukey-Kramer Post-hoc test **(b, d)**, Kruskal-Wallis test with Dunn’s Post-hoc test **(f)**, Wilcoxon signed-rank test (**f**, 18 to 20 months), paired *t*-test (**b**, 12 vs 14 months). *** P < 0.001, **** P < 0.0001

The differences in plaque densities, sizes and growth rates between the young and old age cohorts are summarized in Figure 3. Mean plaque density started very low in the young cohort at 26.2 mm^-3^ (13.8-49.8 mm^-3^ CI), but increased to 42.0 mm^-3^ (23.2-75.7 mm^-3^ CI) during the 2-month observation period (Figure 3a,b). This robust increase in plaque density was attributed to 50 newly formed plaques that were added to the 101 already-existing plaques at the beginning of the study. In contrast, within the old cohort only 3 new plaques were observed alongside 786 preexisting plaques. As expected, mean plaque density was significantly higher at 18 months of age (191 mm^-3^; 126–292 mm^-3^ CI) compared to 12 and 14 months of age (Figure 3b). Long-term imaging revealed no further significant increase in mean plaque density, which peaked at a plateau of 160 mm^-3^ (100–255 mm^-3^ CI) at 24 months of age (Figure 3c, solid line).

The size of a plaque can be represented by its radius, calculated from the measured volume assuming a spherical plaque shape to facilitate comparisons [19]. At 12 months, newly-formed plaques had a mean radius of 2.27 μm (2.12-2.43 μm CI; Figure 3d), while the mean radius of preexisting plaques was 5.08 μm (4.45-5.80 μm CI). With ageing, mean plaque radii increased further and reached 7.98 μm at 14 months (7.31-8.72 μm CI), 10.8 μm at 18 months (10.4-11.2 μm CI) and 16.7 μm at 27 months (Figure 3d, 15.0-18.6 μm CI). Radii from newly formed plaques showed the narrowest spectrum and were exclusively normally distributed, while sizes from preexisting plaques covered a wide range and had a log-normal distribution (Figure 3e).

A linear regression of plaque radii over time revealed linear growth rates reflected by the slope of the lines [19]. Nascent plaques exhibited the fastest median growth rate at 0.475 μm/week (Figure 3f, 0.326-0.589 μm/week interquartile range, IQR). Preexisting plaques from the young cohort grew at almost the same rate (0.326 μm/week, 0.217-0.479 μm/week IQR; Figure 3f), while the old cohort had the slowest growth rate (0.099 μm/week, -0.070-0.262 μm/week IQR; Figure 3f). Nevertheless, an overall net growth could be detected even in the old cohort (Figure 3g).

**Figure 4:**
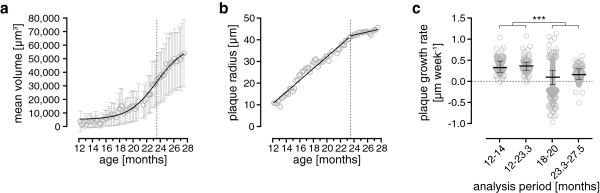
**Bifid analysis of the plaque growth data of long-term imaging. (a)** Mean volume over time (error bars, 95% CI). A sigmoid function was fitted to the mean volume (black line). The dotted line indicates the inflection point of the fitted curve dividing the cubic and asymptotic parts of the function. **(b)** Kinetics of plaque radii. According to the cubic and asymptotic phase of volume growth two linear regressions (black lines) were fit from 12 to 23.3 and 23.5 to 27.5 months of age. This analysis was done for each single plaque. **(c)** Comparison of the plaque growth rates resulting from the bifid analysis with the values gained from the young and old imaging cohort. Black lines with error bars show medians with interquartile range. Multiple comparisons were performed by Kruskal-Wallis test with Dunn’s Post-hoc test. ***P < 0.001

As the long-term data over 15.5 months displayed both phases (cubic and asymptotic) of growth a bifid analysis of the growth rates was performed to compare them to the young and old cohorts (Figure 4a). The cubic and asymptotic growth phases are divided by the inflection point of the fitted sigmoid function that lies at the age of 23.5 months. Therefore, plaque growth rates were determined from 12 to 23.3 and from 23.5 to 27.5 months of age for each single plaque (Figure 4b). The linear plaque growth rate was fastest in the cubic phase (median 0.365 μm/week, 0.258-0.453 μm/week IQR), whereas a strong decline was observed for the asymptotic phase (Figure 4c, median 0.159, 0.049-0.297 μm/week IQR).

### Characteristic relationships between quantitative plaque parameters

**Figure 5:**
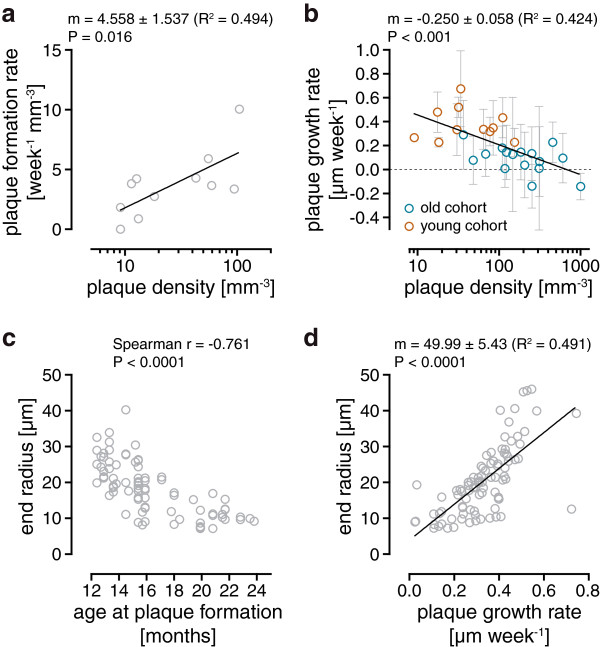
**Correlations between quantitative parameters of plaque growth dynamics. (a)** Plaque formation rate as a function of plaque density. Black line, linear regression (slope is significantly different from zero, p = 0.016). Each circle represents one imaged position. **(b)** Plaque growth rate as a function of plaque density. Error bars show the 95% CI. Black line shows a linear regression of the data. The slope is statistically significant different from zero (p < 0.001). **(c)** Radius of newly formed plaques at the end of long-term imaging over 15.5 months as a function of the age of the mouse at plaque formation (N = 81 newly formed plaques from 2 positions). **(d)** Plaque radius at the end of long-term imaging over 15.5 months as a function of plaque growth rate (N = 90 newly formed and preexisting plaques). Black line shows a linear regression of the data. The slope is statistically significant different from zero. Statistical tests: F-test **(a, b, d)**, Spearman correlation **(c)**.

Relationships between plaque densities, growth rates, size and age at plaque formation were analyzed to enable a detailed characterization of plaque formation and growth. In the early stage of plaque pathology (young cohort) plaque density at the start of imaging correlated with plaque formation rate, the latter being an indicator of the number of plaques formed per volume and time unit (Figure 5a). The average plaque formation rate in the young cohort amounted to 3.40 week^-1^ mm^-3^ (1.63-5.16 week^-1^ mm^-3^ CI). A global comparison of all data collected in the young and old cohort revealed an inverse correlation between averaged plaque growth rates and plaque densities (Figure 5b). In addition, analysis of a single plaque over a period of 15.5 months covering the dynamic cubic and the asymptotic phases of plaque development provided further insight into plaque growth characteristics. The largest plaques at the end of imaging had formed early during plaque development, which leads to a strong inverse correlation between plaque radius and age of the mouse at plaque formation (Figure 5c). Moreover, plaque radius at the end of imaging correlated strongly with the corresponding growth rates of these plaques (Figure 5d).

**Figure 6:**
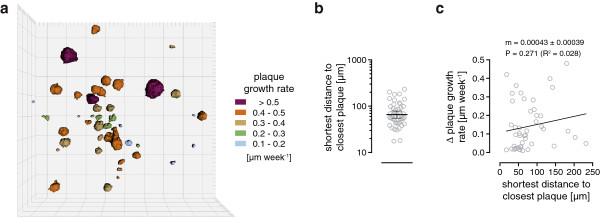
**Spatial relationship between growth rates of neighboring plaques. (a)** Amyloid plaques are displayed as 3D volume-rendered images. Plaque growth rates are color-coded according to a classification into 5 bins. One square represents 100 μm. **(b)** Shortest distance to the closest neighboring plaque (90 plaques), calculated from the long-term imaging over 15.5 months. The shortest distances are displayed in the graph with the mean and 95% CI (65.8 μm, 55.3-78.4 μm CI). **(c)** For each pair of nearest neighbor plaques the difference between their linear plaque growth rates and their shortest distance were calculated (open circles). No relationship between both parameters could be measured by linear regression. The slope was not statistically significant different from zero. Statistical test: F-test **(c)**.

There was a wide distribution of individual plaque growth rates which lead to the question whether there is a specific spatial relationship between growth rates of neighboring plaques. This analysis was performed using data from long-term imaging, the main reason being that a longer observation period facilitates more precise measurements of plaque growth rates. For a visual impression, plaques from overview image of Figure 1b were categorized in five classes according their growth rates. These plaques were displayed as 3D surface-rendered objects with their corresponding growth rate class coded in different colors (Figure 6a). Analysis involved calculating the shortest Euclidean distance of a plaque to its nearest neighbor, which averaged 65.8 μm (55.3-78.4 μm CI; Figure 6b). In the case of a spatial relationship, growth rates of neighboring plaques would be expected to be similar. The analysis of two imaged positions and 46 pairs of plaques (90 plaques total), however, revealed no such relationship (Figure 6c). Moreover, the differences between growth rates of neighboring plaques showed a wide distribution and reflect the visual impression from Figure 6a.

## Discussion

Novel insights into plaque growth kinetics arose from several *in vivo* studies in recent years. Assessment of basic plaque growth parameters in the present study gave results consistent with the majority of findings from the literature. The most prominent feature is the increase of plaque size during ageing [16-20,31]. Plaques in their nascent stage were smallest and constituted a distinct size class that showed a normal distribution. Preexisting plaques were much larger, depending on the age of the mice, and displayed a log-normal distribution as also found in humans [32]. In contrast, the rate of plaque growth decreased with ageing [16,17,20,22]. Several reasons could be responsible for the decline in plaque growth: (i) Aβ production becomes rate limiting, because an ever-increasing total surface area of β-amyloid plaques requires an increase in the amount of Aβ to maintain a constant linear growth rate. (ii) The dissociation of Aβ aggregates from β-amyloid plaques increases. (iii) The number and volume of microglia around plaques increases and changes plaque maintenance [33]. In addition, the arguments (ii) and (iii) may explain negative plaque growth rates in aged animals, whereas we can not completely exclude that at least in part data noise is responsible for negative values. A recent study proposed clustering of plaques as different mechanism of plaque growth [34]. In some rare instances we also observed fusion of plaques which we found as a consequence of their volume growth in combination with close proximity to each other (see image series Figures 1 and 3, Additional file 1). Furthermore, two studies found no steady plaque growth [17,19,21] or plaque growth and shrinkage [33] in the early phase of plaque development. Possible reasons for the inconsistent findings are discussed within the literature and beyond the scope of this publication [16-20].

For the first time we were able to chart the entire kinetics of growth of a single plaque, and demonstrate a sigmoidal growth curve comprising both a cubic and an asymptotic phase (Figures 1 and 2, Additional file 2: Figure S1a-b). Recent studies in humans using positron emission tomography (PET) applying an amyloid tracer (Pittsburgh compound B, PiB) [10,35,36] also found a plateau and/or a sigmoid-shaped progression of β-amyloid accumulation [7,13].

Long-term imaging over at least 6 months revealed an asymptotic increase in plaque densities that could be fitted to a one-phase association function. This finding is congruent with a previously described decline in the rate of plaque formation with ageing [16-20,31]. Interestingly, plaque growth and plaque density are inter-related, as shown by correlation analysis of both parameters.

During the dynamic phase of plaque development (young cohort) a distinct correlation between plaque density and plaque formation rate was observed. In humans, a similar relationship was found using PiB-PET which revealed a higher increase in PiB retention in the second PET scan after 18 months if those subjects had a positive PiB signal in the first PET scan [37]. Fibrillization of Aβ has been convincingly shown by *in vitro* studies to consist of a two-step process that requires first the nucleation of a seed, before polymerization into amyloid fibrils [14,38]. Furthermore, a direct relationship was measured *in vitro* between Aβ concentration and the ability to form nucleation seeds [8,39]. For a mouse model of AD, a similar correlation between Aβ concentration in the interstitial/cerebrospinal fluid and plaque load (a relative measure of the area of a brain slice that is covered by plaques) was reported [40,41]. Taking together the knowledge from these studies with our results, a link between plaque density and Aβ production can be established since there is a strong correlation between plaque density and plaque load (Additional file 2: Figure S1c). Interestingly, plaque densities varied over a wide range which suggests that Aβ production varies between individual Tg2576 mice a fact that was already reported [42]. A possible reason could be the mixed genetic background of Tg2576 mice which may be lead to differences in epigenetic regulation and/or promotor activities [9]. Nevertheless, all 4 animals of the young cohort covered the complete range of plaque densities measured in 30 animals of a further study (Additional file 2: Figure S1d). In addition, the predictive value of plaque density regarding Aβ production could be limited in mouse models without a constant Aβ production over time [23]. Another limitation worth noting is that methoxy-X04 in mice, and also PiB in humans, detect insoluble fibrillar but not soluble Aβ species [29,36,43,44]. Furthermore, *in vivo* two-photon imaging using methoxy-X04 allows for the resolution of single amyloid plaques in brain regions accessible by this technique while PET imaging allows to image the whole brain, but with a resolution limit of about 1 mm^3^.

The observation of a steady linear increase in plaque radius over several months during the cubic growth phase presumes that Aβ concentration is not rate limiting. Otherwise a decline in growth would be expected due to the ever-increasing surface area of β-amyloid plaques which requires a proportional increase in the amount of Aβ to maintain a constant linear growth rate. A comparison of plaque growth of the Tg2576 with the APPPS1 mouse model that was investigated by two other studies, using exactly the same methods and data analysis, supports this hypothetical assumption [16-20,31]. Although APPPS1 mice produce much more Aβ and accumulation starts much earlier than in Tg2576 mice [9,19,23,30] median plaque growth rates were about 0.3 μm/week in both mouse models [16-20,31]. In conclusion, there appears to be an upper limit for median plaque growth *in vivo* that was also identified *in vitro* for the growth of Aβ fibrils [45]. The higher Aβ production in APPPS1 mice causes a much higher plaque density compared to Tg2576 mice (631 mm^-3^, 521–762 mm^-3^ CI vs. 26.2 mm^-3^, 13.8-49.8 mm^-3^ CI, Additional file 2: Figure S1e, Figure 3b). Accordingly, a 10-fold higher rate of plaque formation was reported for APPPS1 mice [19] compared to Tg2576 mice (35 vs. 3.40 week^-1^ mm^-3^). Although median plaque growth rates were similar in both mouse models, individual plaque growth rates showed a big variance. In addition, plaque growth rates of neighboring plaques did not correlate. Combining both results, it is most likely that different conditions within the microenvironment of plaques are causal for the different growth rates. Such conditions could include pH, ionic strength and hydrophobicity of the Aβ peptide, as well as interactions with membranes [46-49]. Even though the analysis of a single animal is statistically not sufficient, the unique dataset containing a complete plaque growth kinetic further supports this idea. It highlights that large plaques were formed early during Aβ accumulation and have the highest growth rates. This in turn suggests that plaques are first formed at sites where the best conditions exist.

**Figure 7:**
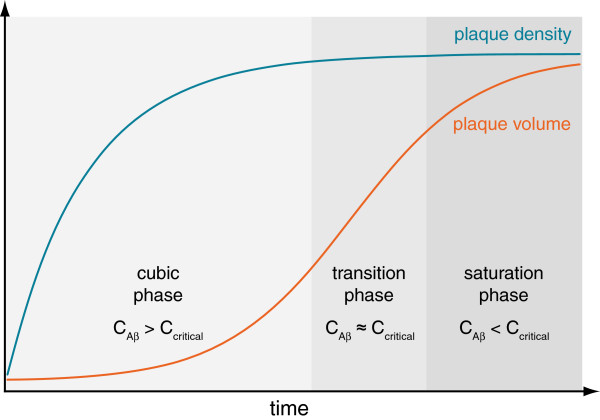
**Model for the relationship between plaque density kinetics and amyloid plaque growth *in vivo.*** The term c_critical_ refers to the minimum critical concentration of Aβ that is necessary to form plaque seeds (a prerequisite discovered by in vitro studies). This initial step of plaque formation was observed by recording the plaque density kinetics *in vivo*. According to the plaque density kinetics and applying the aforementioned requirement different relations of the Aβ concentrations comparative to the critical minimum concentration can be assigned to the different growth phases.

In conclusion, the comprehensive plaque density and growth kinetics point to a three-stage model of β-amyloid accumulation (Figure 7). According to the kinetics of plaque volume three phases can be distinguished: (1) a cubic (2) a transition and (3) a saturation phase. Initially, a high concentration of Aβ above the critical concentration leads to the formation of many new plaques (cubic phase). Later, the large number of plaques may serve as “dumps” for free Aβ, so that its concentration gradually drops, causing the formation of fewer plaques during the transition phase, while existing plaques continue growing. In the saturation phase, single plaques show an overall slower growth due to the large number of plaques, while no new plaques are deposited, thus Aβ production becomes rate-limiting. Furthermore, due to the same sigmoid-shaped characteristic of plaque accumulation in humans, albeit over one to two decades, such a model may help extrapolate observations from AD mouse models to the situation in humans, particularly with regards to pre-clinical testing of Aβ-lowering therapeutics [7,13]. In particular, the determination of plaque density kinetics may be more sensitive than analyzing plaque volume kinetics when evaluating anti-aggregation agents, since Aβ concentration is not rate-limiting to plaque growth in the early stage of amyloid deposition.

## Competing interests

The authors declare that they have no competing interests.

## Supplementary Material

Additional file 1 **This video shows the complete plaque growth kinetic from 12 to 27.5 months of age (image data from Figure** 1**b).** Data is displayed as 3D-volume rendered images. Big squares represent 100 μm. Click here for file

Additional file 2: Figure S1 (a) Plaque volume data of two additional mice over 6 months. Mean plaque volumes were best fit with either a sigmoid function (mouse1: open triangles) or a cubic function (mouse2: open circles). Error bars show 95% CI. (b) Kinetics of the plaque densities (dotted lines) and mean volumes (colored lines showing nonlinear fitted functions) over time for all 3 mice imaged at least over a period of 6 months. Vertical dotted lines indicate time points when plaque densities did not further increase. (c) Relationship of amyloid plaque load (integrated plaque volume/imaging volume) and plaque density for each imaged position. Linear regression was performed for each age cohort. The slopes were statistically significant different from zero (F-test). (d) Comparison of the plaque densities between the young cohort of the current study and another group of mice of a further study. Line and error bars display mean with 95% CI. The means were not significant different (*t*-test). (e) Plaque density measured in APPPS1 mice at 4 months of age. Line and error bars display mean with 95% CI. The equations were used for curve fitting. Click here for file

## References

[B1] Duyckaerts C Delatour B Potier M-C Classification and basic pathology of Alzheimer disease Acta Neuropathol 2009 2 1 5 36 10.1007/s00401-009-0532-1 19381658

[B2] Nalivaeva NN Turner AJ The amyloid precursor protein: a biochemical enigma in brain development, function and disease FEBS Lett 2013 2 13 2046 2054 doi:10.1016/j.febslet.2013.05.010 10.1016/j.febslet.2013.05.010 23684647

[B3] Glenner GG Wong CW Alzheimer's disease: initial report of the purification and characterization of a novel cerebrovascular amyloid protein Biochem Biophys Res Commun 1984 2 3 885 890 10.1016/S0006-291X(84)80190-4 6375662

[B4] Haass C Take five-BACE and the γ-secretase quartet conduct Alzheimer's amyloid β-peptide generation EMBO J 2004 2 3 483 488 10.1038/sj.emboj.7600061 14749724 PMC1271800

[B5] Hardy J Allsop D Amyloid deposition as the central event in the aetiology of Alzheimer's disease Trends Pharmacol Sci 1991 2 10 383 388 1763432 10.1016/0165-6147(91)90609-v

[B6] Hardy J Selkoe DJ The amyloid hypothesis of Alzheimer's disease: progress and problems on the road to therapeutics Science 2002 2 5580 353 356 10.1126/science.1072994 12130773

[B7] Bateman RJ Xiong C Benzinger TLS Fagan AM Goate A Fox NC Marcus DS Cairns NJ Xie X Blazey TM Holtzman DM Santacruz A Buckles V Oliver A Moulder K Aisen PS Ghetti B Klunk WE McDade E Martins RN Masters CL Mayeux R Ringman JM Rossor MN Schofield PR Sperling RA Salloway S Morris JC Network DIA Clinical and biomarker changes in dominantly inherited Alzheimer's disease N Engl J Med 2012 2 9 795 804 10.1056/NEJMoa1202753 22784036 PMC3474597

[B8] Hortschansky P Schroeckh V Christopeit T Zandomeneghi G Fändrich M The aggregation kinetics of Alzheimer's β-amyloid peptide is controlled by stochastic nucleation Protein Sci 2005 2 7 1753 1759 10.1110/ps.041266605 15937275 PMC2253354

[B9] Hsiao K Chapman P Nilsen S Eckman C Harigaya Y Younkin S Yang F Cole G Correlative memory deficits, Aβ elevation, and amyloid plaques in transgenic mice Science 1996 2 5284 99 102 10.1126/science.274.5284.99 8810256

[B10] Nordberg A PET imaging of amyloid in Alzheimer's disease Lancet Neurol 2004 2 9 519 527 doi:10.1016/S1474-4422(04)00853-1 10.1016/S1474-4422(04)00853-1 15324720

[B11] Kang J Lemaire HG Unterbeck A Salbaum JM Masters CL Grzeschik KH Multhaup G Beyreuther K Müller-Hill B The precursor of Alzheimer's disease amyloid A4 protein resembles a cell-surface receptor Nature 1987 2 6106 733 736 10.1038/325733a0 2881207

[B12] Burdick D Soreghan B Kwon M Kosmoski J Knauer M Henschen A Yates J Cotman C Glabe C Assembly and aggregation properties of synthetic Alzheimer's A4/β amyloid peptide analogs J Biol Chem 1992 2 1 546 554 1730616

[B13] Jack CR Jr Wiste HJ Lesnick TG Weigand SD Knopman DS Vemuri P Pankratz VS Senjem ML Gunter JL Mielke MM Lowe VJ Boeve BF Petersen RC Brain β-amyloid load approaches a plateau Neurology 2013 2 10 890 896 Doi:10.1212/WNL.0b013e3182840bbe 10.1212/WNL.0b013e3182840bbe 23446680 PMC3653215

[B14] Jarrett JT Lansbury JPT Seeding "one-dimensional crystallization" of amyloid: a pathogenic mechanism in Alzheimer's disease and scrapie? Cell 1993 2 6 1055 1058 10.1016/0092-8674(93)90635-4 8513491

[B15] Liebscher S Meyer-Luehmann M A peephole into the brain: Neuropathological features of Alzheimer's disease revealed by in vivo two-photon imaging Front Psychiatry 2012 2 26 26 22485096 10.3389/fpsyt.2012.00026 PMC3317174

[B16] Bittner T Burgold S Dorostkar MM Fuhrmann M Wegenast-Braun BM Schmidt B Kretzschmar H Herms J Amyloid plaque formation precedes dendritic spine loss Acta Neuropathol 2012 2 6 797 807 10.1007/s00401-012-1047-8 22993126 PMC3508278

[B17] Burgold S Bittner T Dorostkar MM Kieser D Fuhrmann M Mitteregger G Kretzschmar H Schmidt B Herms J In vivo multiphoton imaging reveals gradual growth of newborn amyloid plaques over weeks Acta Neuropathol 2011 2 3 327 335 10.1007/s00401-010-0787-6 21136067 PMC3038220

[B18] Condello C Schain A Grutzendler J Multicolor time-stamp reveals the dynamics and toxicity of amyloid deposition Sci Rep 2011 2 19 19 22355538 10.1038/srep00019 PMC3216507

[B19] Hefendehl JK Wegenast-Braun BM Liebig C Eicke D Milford D Calhoun ME Kohsaka S Eichner M Jucker M Long-term in vivo imaging of β-amyloid plaque appearance and growth in a mouse model of cerebral β-amyloidosis J Neurosci 2011 2 2 624 629 10.1523/JNEUROSCI.5147-10.2011 21228171 PMC6623424

[B20] Yan P Bero AW Cirrito JR Xiao Q Hu X Wang Y Gonzales E Holtzman DM Lee J-M Characterizing the Appearance and Growth of Amyloid Plaques in APP/PS1 Mice J Neurosci 2009 2 34 10706 10714 10.1523/JNEUROSCI.2637-09.2009 19710322 PMC2756291

[B21] Meyer-Luehmann M Spires-Jones TL Prada C Garcia-Alloza M de Calignon A Rozkalne A Koenigsknecht-Talboo J Holtzman DM Bacskai BJ Hyman BT Rapid appearance and local toxicity of amyloid-β plaques in a mouse model of Alzheimer's disease Nature 2008 2 7179 720 724 10.1038/nature06616 18256671 PMC3264491

[B22] Christie RH Bacskai BJ Zipfel WR Williams RM Kajdasz ST Webb WW Hyman BT Growth arrest of individual senile plaques in a model of Alzheimer's disease observed by in vivo multiphoton microscopy J Neurosci 2001 2 3 858 864 11157072 10.1523/JNEUROSCI.21-03-00858.2001 PMC6762315

[B23] Radde R Bolmont T Kaeser SA Coomaraswamy J Lindau D Stoltze L Calhoun ME Jäggi F Wolburg H Gengler S Haass C Ghetti B Czech C Hölscher C Mathews PM Jucker M Aβ42-driven cerebral amyloidosis in transgenic mice reveals early and robust pathology EMBO Rep 2006 2 9 940 946 10.1038/sj.embor.7400784 16906128 PMC1559665

[B24] Bittner T Fuhrmann M Burgold S Jung CK Volbracht C Steiner H Mitteregger G Kretzschmar HA Haass C Herms J γ-secretase inhibition reduces spine density in vivo via an amyloid precursor protein-dependent pathway J Neurosci 2009 2 33 10405 10409 doi:10.1523/JNEUROSCI.2288-09.2009 10.1523/JNEUROSCI.2288-09.2009 19692615 PMC6665795

[B25] Bittner T Fuhrmann M Burgold S Ochs SM Hoffmann N Mitteregger G Kretzschmar H LaFerla FM Herms J Multiple events lead to dendritic spine loss in triple transgenic Alzheimer's disease mice PLoS One 2010 2 11 e15477 doi:10.1371/journal.pone.0015477 10.1371/journal.pone.0015477 21103384 PMC2982845

[B26] Fuhrmann M Bittner T Jung CK Burgold S Page RM Mitteregger G Haass C LaFerla FM Kretzschmar H Herms J Microglial Cx3cr1 knockout prevents neuron loss in a mouse model of Alzheimer's disease Nat Neurosci 2010 2 4 411 413 doi:10.1038/nn.2511 10.1038/nn.2511 20305648 PMC4072212

[B27] Fuhrmann M Mitteregger G Kretzschmar H Herms J Dendritic pathology in prion disease starts at the synaptic spine J Neurosci 2007 2 23 6224 6233 10.1523/JNEUROSCI.5062-06.2007 17553995 PMC6672160

[B28] Holtmaat A Bonhoeffer T Chow DK Chuckowree J Paola VD Hofer SB Hübener M Keck T Knott G Lee W-CA Mostany R Mrsic-Flogel TD Nedivi E Portera-Cailliau C Svoboda K Trachtenberg JT Wilbrecht L Long-term, high-resolution imaging in the mouse neocortex through a chronic cranial window Nat Protoc 2009 2 8 1128 1144 10.1038/nprot.2009.89 19617885 PMC3072839

[B29] Klunk WE Bacskai BJ Mathis CA Kajdasz ST McLellan ME Frosch MP Debnath ML Holt DP Wang Y Hyman BT Imaging Aβ plaques in living transgenic mice with multiphoton microscopy and methoxy-X04, a systemically administered Congo red derivative J Neuropathol Exp Neurol 2002 2 9 797 805 12230326 10.1093/jnen/61.9.797

[B30] Das P Verbeeck C Minter L Chakrabarty P Felsenstein K Kukar T Maharvi G Fauq A Osborne BA Golde TE Transient pharmacologic lowering of Aβ production prior to deposition results in sustained reduction of amyloid plaque pathology Mol Neurodegener 2012 2 39 1 9 22892055 10.1186/1750-1326-7-39 PMC3477045

[B31] Crowe SE Ellis-Davies GC In vivo characterization of a bigenic fluorescent mouse model of Alzheimer's disease with neurodegeneration J Comp Neurol 2013 2 10 2181 2194 doi:10.1002/cne.23306 10.1002/cne.23306 23348594 PMC4134262

[B32] Hyman BT West HL Rebeck GW Buldyrev SV Mantegna RN Ukleja M Havlin S Stanley HE Quantitative analysis of senile plaques in Alzheimer disease: observation of log-normal size distribution and molecular epidemiology of differences associated with apolipoprotein E genotype and trisomy 21 (Down syndrome) Proc Natl Acad Sci U S A 1995 2 8 3586 3590 10.1073/pnas.92.8.3586 7724603 PMC42212

[B33] Bolmont T Haiss F Eicke D Radde R Mathis CA Klunk WE Kohsaka S Jucker M Calhoun ME Dynamics of the microglial/amyloid interaction indicate a role in plaque maintenance J Neurosci 2008 2 16 4283 4292 doi:10.1523/jneurosci.4814-07.2008 10.1523/JNEUROSCI.4814-07.2008 18417708 PMC3844768

[B34] McCarter JF Liebscher S Bachhuber T Abou-Ajram C Hubener M Hyman BT Haass C Meyer-Luehmann M Clustering of plaques contributes to plaque growth in a mouse model of Alzheimer's disease Acta Neuropathol 2013 2 2 179 188 doi:10.1007/s00401-013-1137-2 10.1007/s00401-013-1137-2 23775142 PMC3722456

[B35] Mathis CA Wang Y Holt DP Huang GF Debnath ML Klunk WE Synthesis and evaluation of 11C-labeled 6-substituted 2-arylbenzothiazoles as amyloid imaging agents J Med Chem 2003 2 13 2740 2754 doi:10.1021/jm030026b 10.1021/jm030026b 12801237

[B36] Mori T Maeda J Shimada H Higuchi M Shinotoh H Ueno S Suhara T Molecular imaging of dementia Psychogeriatrics 2012 2 2 106 114 doi:10.1111/j.1479-8301.2012.00409.x 10.1111/j.1479-8301.2012.00409.x 22712644

[B37] Villain N Chetelat G Grassiot B Bourgeat P Jones G Ellis KA Ames D Martins RN Eustache F Salvado O Masters CL Rowe CC Villemagne VL Regional dynamics of amyloid-β deposition in healthy elderly, mild cognitive impairment and Alzheimer's disease: a voxelwise PiB-PET longitudinal study Brain 2012 2 Pt 7 2126 2139 doi:10.1093/brain/aws125 22628162 10.1093/brain/aws125

[B38] Harper JD Lansbury PT Models of amyloid seeding in Alzheimer's disease and scrapie: mechanistic truths and physiological consequences of the time-dependent solubility of amyloid proteins Annu Rev Biochem 1997 2 385 407 10.1146/annurev.biochem.66.1.385 9242912

[B39] Hellstrand E Boland B Walsh DM Linse S Amyloid β-Protein Aggregation Produces Highly Reproducible Kinetic Data and Occurs by a Two-Phase Process ACS Chem Neurosci 2010 2 1 13 18 10.1021/cn900015v 22778803 PMC3368626

[B40] Cirrito JR May PC O'Dell MA Taylor JW Parsadanian M Cramer JW Audia JE Nissen JS Bales KR Paul SM DeMattos RB Holtzman DM In vivo assessment of brain interstitial fluid with microdialysis reveals plaque-associated changes in amyloid-β metabolism and half-life J Neurosci 2003 2 26 8844 8853 14523085 10.1523/JNEUROSCI.23-26-08844.2003 PMC6740389

[B41] DeMattos RB Bales KR Parsadanian M O'Dell MA Foss EM Paul SM Holtzman DM Plaque-associated disruption of CSF and plasma amyloid-β (Aβ) equilibrium in a mouse model of Alzheimer's disease J Neurochem 2002 2 2 229 236 10.1046/j.1471-4159.2002.00889.x 12064470

[B42] Taconic Biochemical Characterization Alzheimer's Disease Modelshttp://www.taconic.com/1349. (Accessed 10/04/2014)

[B43] Cairns NJ Ikonomovic MD Benzinger T Storandt M Fagan AM Shah AR Reinwald LT Carter D Felton A Holtzman DM Mintun MA Klunk WE Morris JC Absence of Pittsburgh compound B detection of cerebral amyloid beta in a patient with clinical, cognitive, and cerebrospinal fluid markers of Alzheimer disease: a case report Arch Neurol 2009 2 12 1557 1562 doi:10.1001/archneurol.2009.279 20008664 10.1001/archneurol.2009.279 PMC2796200

[B44] Ikonomovic MD Klunk WE Abrahamson EE Mathis CA Price JC Tsopelas ND Lopresti BJ Ziolko S Bi W Paljug WR Debnath ML Hope CE Isanski BA Hamilton RL DeKosky ST Post-mortem correlates of in vivo PiB-PET amyloid imaging in a typical case of Alzheimer's disease Brain 2008 2 Pt 6 1630 1645 doi:10.1093/brain/awn016 18339640 10.1093/brain/awn016 PMC2408940

[B45] Lomakin A Chung DS Benedek GB Kirschner DA Teplow DB On the nucleation and growth of amyloid β-protein fibrils: detection of nuclei and quantitation of rate constants Proc Natl Acad Sci U S A 1996 2 3 1125 1129 10.1073/pnas.93.3.1125 8577726 PMC40042

[B46] Chiti F Stefani M Taddei N Ramponi G Dobson CM Rationalization of the effects of mutations on peptide and protein aggregation rates Nature 2003 2 6950 805 808 10.1038/nature01891 12917692

[B47] DuBay KF Pawar AP Chiti F Zurdo J Dobson CM Vendruscolo M Prediction of the absolute aggregation rates of amyloidogenic polypeptide chains J Mol Biol 2004 2 5 1317 1326 10.1016/j.jmb.2004.06.043 15302561

[B48] Esler WP Stimson ER Ghilardi JR Vinters HV Lee JP Mantyh PW Maggio JE In vitro growth of Alzheimer's disease β-amyloid plaques displays first-order kinetics Biochemistry 1996 2 3 749 757 10.1021/bi951685w 8547255

[B49] Terzi E Hölzemann G Seelig J Self-association of β-amyloid peptide (1–40) in solution and binding to lipid membranes J Mol Biol 1995 2 5 633 642 10.1006/jmbi.1995.0525 7563079

